# Information Across the Ecological Hierarchy

**DOI:** 10.3390/e21100949

**Published:** 2019-09-27

**Authors:** Robert E. Ulanowicz

**Affiliations:** 1 Department of Biology, University of Florida, Gainesville, FL 32611-8525, USA; ulan@umces.edu; Tel.: +1-352-378-7355; 2 Center for Environmental Science, University of Maryland, Solomons, MD 20688-0038, USA

**Keywords:** centripetality, conditional entropy, entropy, information, MAXENT, mutual information, network analysis

## Abstract

The ecosystem is a theatre upon which is presented, in various degrees and at differing scales, a drama of constraint and information vs. disorganization and entropy. Concerning biology, most think immediately of genomic information. It strongly constrains the form and behavior of individual species, but its influence upon community structure is indeterminate. At the community level, information acts as a formal cause behind regular patterns of development. Community structure is an amalgam of information and entropy, and the Gibbs–Boltzmann formula departs from the thermodynamic sense of entropy. It measures only the extreme that entropy might reach if the elements of the system were completely independent. A closer analogy to physical entropy in systems with interactions is the conditional entropy—the amount by which the Shannon measure is reduced after the information in the constraints among elements has been subtracted. Finally, at the whole ecosystem level, in communities that inhabit mostly fixed physical environments (e.g., landscapes or seabeds), the distributions of plants and animals appear to be independent both of causal mechanisms and trophic controls, and assume instead forms that maximize the overall entropy of dispersal.

## 1. Genomic Information Acts Primarily on Organisms

Information plays different roles in ecology at various scales, and the goal here is to characterize and compare those disparate actions. Attention to information among contemporary biologists is primarily devoted to the influence of the genome, and such preoccupation elicits among many ecologists an image of the aggregate gene pool determining community structure. Of course, the genetic composition of a species does exert dramatic influence upon the autecology of a given species, however, it remains ambiguous, whether the secondary consequences of those effects give rise to the dynamical order of the entire system (synecology). Consider, for example, the words of renowned developmental biologist, Gunter Stent:

Consider the establishment of ecological communities upon colonization of islands or the growth of secondary forests. Both of these examples are regular phenomena in the sense that a more or less predictable ecological structure arises via a stereotypic pattern of intermediate steps, in which the relative abundances of various types of flora and fauna follow a well-defined sequence. The regularity of these phenomena is obviously not the consequence of an ecological program encoded in the genomes of the participating taxa [1].

Stent is inferring that events at the level of the ecological community and the larger environment are shaping the dynamics and composition of the system. Recent research to the contrary has recorded how the downstream effects of genomic changes can be comparable to, or even greater than, that of direct species replacement [2]. Even more recent thinking, however, emphasizes how the possibilities pursuant to such changes cannot even be formulated in advance [3,4], so that, effectively, the role of genomes appears to be limited to recording the success or failure of their particular phenotypes and their associated behaviors.

## 2. System Level Information

As developmental biologist Sidney Brenner exhorted biologists, “we have to discover the principles of organization, how lots of things are put together in the same place” [1]. This ambition pertains to the level of synecology, which at first glance may seem to have little to do with information. However, it was only 7 years after Shannon’s [5] inception of contemporary information theory that Robert MacArthur [6] used the Shannon diversity index to quantify the variety of transfer magnitudes among entire networks of trophic flows. MacArthur was promoting the idea that such diversity is somehow correlated with overall functional redundancy, which could help to stabilize a system.

Unfortunately, measuring flows is a laborious endeavor, and attention soon shifted to using the Shannon index to quantify the diversity of biomass densities or population numbers among the components of an ecosystem—a quantity that became known as “biocomplexity”. The search for a connection between biocomplexity and system stability preoccupied theoretical ecology during the 1960s. In the process, a number of other formulations for system diversity appeared [7,8], but none possessed the attribute of additivity, which allows for the ready decomposition of an entropy-like metric. Subsequent investigations have revealed that biocomplexity does not correlate at all well with functional diversity [9].

It was several years after Robert May [10] demonstrated how more species could imply less stability that Rutledge et al. [11] reconsidered MacArthur’s emphasis on flows, and introduced conditional probabilities into the information narrative. MacArthur had originally characterized the distribution of flows in ecosystems as,

(1) $$D=-\sum_{k}p_{k}\;\log\left(p_{k}\right)$$

where *p_k_* is the probability of a quantum of total medium being part of flow *k*, and *D* is the diversity of flow magnitudes. The problem with MacArthur’s indicial notation is that it neglects the fact that a flow is a relationship between categories, and the endpoints of each relationship (i.e., the predator and the prey involved) must be recognized. So rather than *p_k_*, it is more ecological to focus on *p_ij_* as the probability of a flow from donor *i* to recipient *j* and write,

(2) $$H=-\sum_{i,j}p_{ij}\;\log\left(p_{ij}\right)$$

where *H* is the diversity of the joint interactions of all pairs *i* and *j*.

Shannon named his index “entropy”, because Ludwig Boltzmann and Josiah Willard Gibbs independently derived much the same formula to quantify the entropy, *H*, of a perfect gas. It is important to note, however, that *H* does not represent entropy in general. The atoms of a perfect gas are completely independent of each other. A general metric of entropy would involve system tokens *i* and *j* that interact strongly with one another. When the system under study is an ecosystem, for example, predators and prey are constantly limiting or embellishing one another’s populations, and one must also account for such constraints among system elements. Doing this involves defining the marginal probabilities of inputs and outputs pertaining to each species as $p_{.j}$ = $\sum_{i}p_{ij}$ and $p_{i.}$ = $\sum_{j}p_{ij}$, respectively. The degree to which *i* depends on *j* (and vice versa) will be represented by the conditional probabilities (*p_ij_/p_i._*) and (*p_ij_/p_.j_*), respectively. For example, if predator *j* consumes 100 units of prey *i* per unit time, and the total output of *i* (*p_i._*) is 1000 units/time, then the conditional probability that the next predator to feed on *i* would be *j* is estimated by the frequency (conditional probability, *p_ij_*/*p_i_*_._) as 0.1. Similarly, if the total consumption by predator *j* is 200 units per time, then *p_ij_*/*p_.j_* ~ 0.5.

Nonzero conditional probabilities allow *H* to be decomposed into two separate non-negative terms, *A* and *Φ*,

(3) $$\begin{array}{c} H=A+\Phi \\ -\sum_{i,j}p_{ij}\;\log\left(p_{ij}\right)=\sum_{i,j}p_{ij}\log\left(p_{ij}/p_{i.}p_{.j}\right)-\sum_{i,j}p_{ij}\log\left(p_{ij}^{2}/p_{i.}p_{.j}\right) \end{array}$$

where *A* (≥0) quantifies the fraction of *H* that estimates how constrained or coordinated the components of the system are with one another, and *Φ* (also ≥0) measures the degree to which elements remain independent of each other [11,12]. In information theory, *A* is called the “average mutual information”, and *Φ* the “conditional entropy”. Hence, the conditional entropy, *Φ*, appears to be a general analogy to physical entropy and in ecosystems it typically differs from *H* by 40% or so [13].

To recast *A* and *Φ* in terms of actual measurements or estimates of flows, let *T_ij_* represent the physical flow from *i* to *j*, and, as with the conditional probabilities, a dot in place of a subscript will indicate summation over that index. Then equation (3) translates into

(4) $$\begin{array}{c} H=A+\Phi \\ -\sum_{i,j}\frac{T_{ij}}{T_{..}}\log\left(\frac{T_{ij}}{T_{..}}\right)=\sum_{i,j}\frac{T_{ij}}{T_{..}}\log\left(T_{ij}T_{..}/T_{i.}T_{.j}\right)-\sum_{i,j}\frac{T_{ij}}{T_{..}}\log\left(T_{ij}^{2}/T_{i.}T_{.j}\right) \end{array}$$

The reader will note that (*A/H*) + (*Φ*/*H*) = 1, so that *A/H* represents the fraction of system activity devoted to constrained organized behavior, whereas *Φ*/*H* gauges the fraction that can be characterized as remaining free, disorganized, or flexible.

It was originally thought that *A* → 1 as an ecosystem matures [14], i.e., ecosystems grow increasingly more efficient. As more networks were quantified and catalogued, however, data did not support that assumption. Rather, networks of diverse ecosystems appear to cluster in the interval 0.35 < *A/H* < 0.45, which has been called the “window of vitality” by Ulanowicz [15], who later provided a method for identifying how each flow needs to be changed and by what relative amount so as to move a system towards the center of the window [13]. Recently, the window of vitality has been implemented as a “biomimicry” criterion to introduce fail-safe reliability into the design of power grids [16] and water supply networks [17].

## 3. The Relationship Between Shannon Diversity and True Entropy

How, then, does H in (3) relate to actual physical entropy? Original phenomenological thermodynamics distinguished between the total energy possessed by a system and the fraction thereof that can be converted into work. Conservation of energy is preserved by writing the total energy as the sum of useful energy and otherwise inaccessible energy as the sum,

*U* = *G* + *TS* (5)

where *U* denotes the total energy; *G*, that energy available to do work; *T*, the absolute temperature of the system; *S*, the physical entropy of the system. (*G* is the Gibbs free energy, appropriate under conditions of constant gas pressure. Helmholz also defined a corresponding free energy, *H = A + TS*, pertaining to constant volume situations, but his meanings of *H* and *A* differ from the statistical variables defined in this work, and so only the Gibbs version was used to avoid confusion.)

Now, *G* varies according to the internal structure of the system and is zero for systems in which the particles do not interact (like an ideal gas). Dividing both sides of Equation (5) by the absolute temperature, *T*, yields

*U/T* = *G/T* + *S* (6)

Comparing (6) with (4), reveals that H in (3) is analogous to the energy density (U/T), A to the density of useful energy (G/T), and Φ to the physical entropy (S). Again, for a perfect gas or a system of noninteracting tokens, G/T = 0, so that H, the statistical diversity, is then a sufficient analogy to the physical entropy. However, in most real world systems there exists some degree of constraint among system elements. For example, it has already been mentioned how in ecology significant constraints link system members, so that G/T typically accounts for 40% of U/T [13].

In light of the foregoing considerations, it might be useful to remark on how they might affect the common terminology pertaining to the topic of entropy. First and foremost, it should be kept in mind that information and entropy are strict antonyms. Information is a positivist concept, usually deriving from palpable constraints. Entropy, by stark contrast, is the absence of constraint. It represents something that does not exist—an apophasis. John von Neumann was mostly accurate when he said to Shannon that “nobody really understands entropy”, because it is difficult to wrap one’s mind around something that doesn’t exist [18]. That many still refer to the Gibbs–Helmholz formula both as “information” and “entropy” has been the nexus of considerable confusion over the decades (see Chapter 5 in [19]). Shannon might have done better staying with his notion of “capacity”. Similarly, with all due respect to the considerable genius of Schrödinger, the term “negentropy” identifies matters completely backwards and should be staunchly avoided. Entropy is wrongly portrayed as the positivist element and information is inferred in a negative sense.

Then there is the difference between statistical and thermodynamical entropies. Thermodynamicists were measuring entropies during the 19th century as palpable heat transferred at a particular absolute temperature. There was no mention of particles (this author was taught engineering thermodynamics in a manner where any mention of atoms or molecules was strictly forbidden). Later in that century, Boltzman and Gibbs sought to reconcile phenomenological entropy with statistical theoretical considerations on particle motions. It must be mentioned that this was strictly a mental exercise carried out under extreme assumptions. The medium was an ideal gas within which there were no interactions among the atoms. Stochasticity was introduced as part of the boundary statement, as was the unrealistic ergodic assumption. Such conditions do not pertain to reality, apparently not even conditions in intergalactic space.

Mention of these limitations is not to infer that statistical entropy lacks highly useful applications, however. When such nondimensional mathematical attributes are scaled by physical dimensions, it becomes possible to consider the product as a physical attribute of a system. Thus, when the *A* in (3) is multiplied by the total system throughput *T..*, the result is called the ascendency, a measure of the ability of the system to prevail against other similar systems [14]. Ludovosi and Scharler [20], for example, have demonstrated how increasing ascendency is indicative of systems more dominated by K-strategists. Despite the fact that the conditional entropy, *Φ*, in (3), remains a closer analog to physical entropy, it remains an “ecological state function”, distinct from the physical entropy, which is a “physical state function”. Finally, that *Φ* bears the modifier “conditional” reinforces the much-neglected third law of thermodynamics, which stipulates that entropy can only be measured with respect to a chosen reference point. Entropy is intrinsically conditional (and by inference through (3), information is always relative as well).

## 4. Maximal Entropy in Mature Environments

Obviously, purely mental constructs can serve highly useful purposes. Such is the case with statistical entropy. It was intentionally tailored to pertain to a system entirely without organization. Whence, for example, if one wishes to distribute species across a landscape, one has only partial knowledge of the constraints governing the distribution, and one would like to assign the full distribution without making any further unnecessary inferences, one could complete the distribution by maximizing the statistical entropy of the final distribution (MAXENT). Harte has used this methodology with remarkable success to improve one’s knowledge of what rules may be at play among distributions of species, metabolism, body sizes, or wildfire activity (an epistemological endeavor) [21]. Towards this end, Harte [22] has pioneered the successful application of the MAXENT algorithm (the maximization of *H*) to prognosticate rules for numerous ecological distributions. Harte et al. have nested MAXENT within a larger program, METE (Maximal Entropy Theory of Ecology), which facilitates the inclusion of known overall ecosystem constraints. 

Significant about METE is that success was achieved without having to invoke any ecological mechanisms whatsoever. Harte et al. generally work with terrestrial ecosystems where the matrix of the physical environment (the landscape) usually changes slowly with respect to the dynamics of interspecies interactions (the same could probably be achieved for aquatic or marine ecosystems where species distribute across a more-or-less stationary seabed). Harte suggests (personal communication) that transient dynamics are occurring rapidly enough so that populations have sufficient time to balance with the landscape and themselves, without regard to the connections or mechanisms that affect the resultant distributions.

When the larger environment changes more rapidly, the application of METE becomes problematic [23]. It is likely under such dynamical conditions that trophic exchanges begin to affect distributions. These exchanges are acting in the guise of dynamical constraints embodied in the variable *A* of (3). These constraints diminish the potential freedom represented by *H*, so that only the residual *Φ* remains. Assuming that the thrust of the MAXENT methodology still pertains, a plausible move would now be to replace the maximization of *H* with that of *Φ*. Because *Φ* and *H* are both first-order homogeneous Euler functions in the *T_ij_* [24], such change in objective function should not unduly complicate the Lagrangian multiplier optimization algorithm. Presumably, the constraints embodied in *A* should guide the solution towards distributions that are more consonant with the changing conditions. Whether this protocol will lead to the discovery of dynamical ecological rules in analogy with the equilibrium distributions (laws?) uncovered by maximizing *H* remains the subject of future research.

As noted above, there is a proclivity for ecosystems to cluster around about 40% constraint (*A*/*H*) and 60% freedom (*Φ*/*H*). If, then, one defines *a* = *A*/*H* as the degree of organization, then 0 ≤ *a* ≤ 1, and the location of the window of vitality is near to the maximum of the function −*a*log(*a*), an entropy homolog. This could suggest a connection with the notion of maximal entropy. It is worth noting that *a* represents the existence of organization in the system, whereas −log(*a*) maps isomorphically (one-to-one) into (1−*a*), the complement of organization. The product, −*a*log(*a*), therefore quantifies the joint countervailing tendencies to remain the same and to change, respectively. The fact that various ecosystems plot near the maximum of −*a*log(*a*) invites the speculation that this quantity assesses the robustness of ecosystems to evolve and to change in a conservative way.

The reader will note that, like with the application of MAXENT, the quantitative measures defined in (2)–(6) also require no reference to eliciting mechanisms. Such independence suggests the possibility of a macroscopic theory of ecology that is not determined by particular mechanisms. How could this possibly be?

Certainly, ecological mechanisms are highly restrictive of behaviors within a particular focal level of interest, and their elucidation has handsomely enriched the study of ecology. However, as the scope of the discipline expands, the radius of influence of mechanical constraints likely wanes.

Two possibilities might account for this growing indeterminacy. In science, one usually regards the mapping from mechanisms to their effects as isomorphic. With complex systems, the mapping is likely to become homomorphic (many-to-one) [25]. That is, very different mechanisms could contribute to the same result at the next level up. Secondly, causality is generally assumed to be binary—a single relationship links cause with effect. In complex systems, configurations of relationships can serve as the distributed cause of a phenomenon. For example, autocatalytic interaction among closed pathways of self-reinforcing processes gives rise to the ubiquitous tendency of living systems to accumulate energy and resources, a phenomenon called centripetality [26]. Other explanations are possible. The potential for a burst of creativity in macroecology is enormous.

## 5. Conclusions

When viewed across the hierarchy of living systems, the involvement of information bears similarity to a familiar picture. At the smallest (molecular genome) level, information is dense and strong, although its reach is limited to the domain of the individual organism. Advancing to the community level, information becomes distributed and not as restrictive, but gives rise to sufficient centripetality [26] to maintain recognizable forms. In the widest perspective (the full ecosystem), stable physical environments allow distributions to evolve with maximal freedom, as indicated by their conformity to the principle of maximal entropy.

This succession bears resemblance to the evolution of the cosmos subsequent to the recombination event about 380,000 years after the Big Bang. This was when stable matter began to appear—protons, neutrons, electrons, and simple but stable atoms of hydrogen and helium. Energy in these elements is extremely dense and is held in place by overwhelming constraints (nuclear and electromagnetic forces). With further expansion, matter began to concentrate under the weaker but centripetal influence of gravitation into stars, and their interactions gave rise to galactic forms. At the ultimate level of the full cosmos, spacetime is expanding as galaxies continue to separate at an accelerating rate. The intergalactic statistical entropy of matter continues to maximize.

A familiar trope in biology, attributed to Ernst Haeckel, is that “ontogeny recapitulates phylogeny”. It has also been remarked how the emergence of life recapitulates the appearance of matter in [27] (p. 148). That nature at large is best portrayed as the balance between complementary trends has long been an element of Eastern thought (see, for example, [28]) which has been promoted more recently in Western science [29,30]. It is beginning to appear that the cosmological drama at all levels is a continuous dance between the expansiveness of entropy and the centripetality of self-reinforcing information.

## References

[1] Lewin R. (1984). Why is development so illogical?. Science.

[2] Des Roches S., Post D.M., Turley N.E., Bailey J.K., Hendry A.P., Kinnison M.T., Schweitzer J.A., Palkovacs E.P. (2018). The ecological importance of intraspecific variation. Nat. Ecol. Evol..

[3] Ulanowicz R.E. (2016). Process ecology: Philosophy passes into praxis. Process Stud..

[4] Kauffman S.A. (2019). A World Beyond Physics: The Emergence and Evolution of Life.

[5] Shannon C.E. (1948). A mathematical theory of communication. Bell Syst. Tech. J..

[6] MacArthur R.H. (1955). Fluctuations of animal populations and a measure of community stability. Ecology.

[7] Jost L. (2006). Entropy and diversity. Oikos.

[8] Tsallis C., Brigatti E. (2004). Nonextensive statistical mechanics: A brief introduction. Contin. Mech. Thermodyn..

[9] Ulanowicz R.E. (2018). Biodiversity, functional redundancy and system stability: Subtle connections. J. R. Soc. Interface.

[10] May R.M. (1972). Will a large complex system be stable?. Nature.

[11] Rutledge R.W., Basore B.L., Mulholland R.J. (1976). Ecological stability: An information theory viewpoint. J. Theor. Biol..

[12] Ulanowicz R.E., Norden J.S. (1990). Symmetrical overhead in flow networks. Int. J. Sys. Sci..

[13] Ulanowicz R.E. (2009). The dual nature of ecosystem dynamics. Ecol. Model..

[14] Ulanowicz R.E. (1980). An hypothesis on the development of natural communities. J. Theor. Biol..

[15] Ulanowicz R.E., Rinaldo A., Marani A. (1997). Limitations on the Connectivity of Ecosystem Flow Networks. Biological Models.

[16] Panyam V., Huang H., Pinte B., Davis K., Layton A., Walker J.M. Bio-Inspired Design for Robust Power Networks. Proceedings of the 2019 IEEE Texas Power and Energy Conference (TPEC).

[17] Dave T., Layton A. (2019). Bio-inspired design for resilient water distribution networks. Procedia CIRP.

[18] Tribus M., McIrvine E.C. (1971). Energy and information. Sci. Am..

[19] Ulanowicz R.E. (1986). Growth and Development: Phenomenological Ecology.

[20] Ludovisi A., Scharler U.M. (2017). Towards a sounder interpretation of entropy-based indicators in ecological network analysis. Ecol. Indic..

[21] Brummer A.B., Newman E.A. (2019). Derivations of the core functions of the Maximum Entropy Theory of ecology. Entropy.

[22] Harte J. (2011). Maximum Entropy and Ecology: A Theory of Abundance, Distribution, and Energetics.

[23] Harte J., Newman E.A. (2014). Maximum information entropy: A foundation for ecological theory. Trends Ecol. Evol..

[24] Courant R. (1936). Differential and Integral Calculus.

[25] Frank S.A. (2014). Generative models versus underlying symmetries to explain biological pattern. J. Evol. Biol..

[26] Ulanowicz R.E. (1997). Ecology, the Ascendent Perspective.

[27] Ulanowicz R.E. (2009). A Third Window: Natural Life beyond Newton and Darwin.

[28] Xu Z., Cheng G., Ulanowicz R.E., Song X., Deng X., Zhong F. (2018). The Common Developmental Road: Tensions among Centripetal and Centrifugal Dynamics. Nat. Sci. Rev..

[29] Lupasco S. (1952). Le principe d’antagonisme et la logique de l’énergie. Rev. Métaphysique et de Morale.

[30] Brenner J. (2008). Logic in Reality.

